# Efficient pairwise RNA structure prediction and alignment using sequence alignment constraints

**DOI:** 10.1186/1471-2105-7-400

**Published:** 2006-09-04

**Authors:** Robin D Dowell, Sean R Eddy

**Affiliations:** 1 Howard Hughes Medical Institute and Department of Genetics, Washington University School of Medicine, 4444 Forest Park Blvd. Box 8510, St. Louis, MO 63108, USA; 2 MIT Computer Science and Artificial Intelligence Laboratory, 32 Vassar Street, Cambridge, MA 02139, USA

## Abstract

**Background:**

We are interested in the problem of predicting secondary structure for small sets of homologous RNAs, by incorporating limited comparative sequence information into an RNA folding model. The Sankoff algorithm for simultaneous RNA folding and alignment is a basis for approaches to this problem. There are two open problems in applying a Sankoff algorithm: development of a good unified scoring system for alignment and folding and development of practical heuristics for dealing with the computational complexity of the algorithm.

**Results:**

We use probabilistic models (pair stochastic context-free grammars, pairSCFGs) as a unifying framework for scoring pairwise alignment and folding. A constrained version of the pairSCFG structural alignment algorithm was developed which assumes knowledge of a few confidently aligned positions (pins). These pins are selected based on the posterior probabilities of a probabilistic pairwise sequence alignment.

**Conclusion:**

Pairwise RNA structural alignment improves on structure prediction accuracy relative to single sequence folding. Constraining on alignment is a straightforward method of reducing the runtime and memory requirements of the algorithm. Five practical implementations of the pairwise Sankoff algorithm – this work (Consan), David Mathews' Dynalign, Ian Holmes' Stemloc, Ivo Hofacker's PMcomp, and Jan Gorodkin's FOLDALIGN – have comparable overall performance with different strengths and weaknesses.

## Background

RNA secondary structure can be predicted accurately from sequence data alone. For example, the predicted secondary structure of ribosomal RNA has been essentially confirmed by recent crystal structures; 97–98% of the predicted base pairs are confirmed by experimental structures [1]. The trouble is that rRNA predictions were refined by experts over twenty years, ultimately utilizing data from about 7000 small subunit rRNA sequences and 1050 large subunit rRNA sequences [1]. As there are many RNA structures of biological interest [2,3], it is important to find computational means of accelerating, automating, and improving RNA secondary structure prediction [4].

There are two main sources of information for RNA secondary structure prediction. The most accurate means of prediction is *comparative analysis *[5-7], which uses evolutionary information. Homologous RNAs tend to conserve a common base-paired secondary structure. Important base pairing interactions are conserved by compensatory mutations and compensatory mutations induce detectable pairwise sequence correlations between positions of a multiple alignment of homologous RNAs. Statistical methods are used to detect co-varying base pairs, ranging from mutual information criteria [8,6] to more sophisticated phylogenetic models [9-12]. Given an accurate multiple alignment, a large number of sequences, and sufficient sequence diversity, comparative analysis alone is sufficient to produce accurate structure predictions [1]. The ribosomal RNA secondary structure predictions were derived primarily from comparative analysis.

One also has substantial *a priori *knowledge of how RNAs are likely to fold. RNA structures favor certain base stacking interactions and loop lengths, as a result of the thermodynamics of a folded structure. A nearest-neighbor model for predicting the free energy of RNA secondary structures has been developed [13]. Given an RNA sequence and the thermodynamic model, efficient dynamic programming algorithms exist for finding a minimum free energy secondary structure [14-17,13,4]. Energy minimization is not as accurate as comparative analysis [18,13], but unlike comparative analysis, it can be applied to single RNA sequences.

A problem of current interest is the prediction of a consensus secondary structure for a small number of structurally homologous RNA sequences, i.e. when some evolutionary information is available, but not enough for a pure comparative analysis approach. Here one wants to combine evolutionary information and thermodynamic information. This problem now arises frequently because of the availability of comparative genome sequence data. Approaches described thus far fall into two classes: the RNA sequence alignment is treated as known [19,11-23], or the sequences are given unaligned, leading to an even harder problem of simultaneous folding and alignment [24-33].

Here we are interested in the problem of simultaneous folding and alignment ("structural alignment"), for which an algorithmic solution was described by David Sankoff [24]. The algorithm is computationally demanding, requiring *O*(*L*^2*n*^) and *O*(*L*^3*n*^) in space and time respectively for *n *sequences of length *L*. Consequently, most subsequent work on the problem has focused on the case of pairwise structural alignment, limiting the number of sequences to two (*n *= 2).

When applying a consensus structure prediction algorithm, it is necessary to decide what score should be optimized by the algorithm, so that a mathematically optimal structure has the best chance of representing the biologically correct structure. The evolutionary information in comparative sequence analysis is most naturally treated by probabilistic models. Single-sequence RNA secondary structure predictions are usually scored by thermodynamic models and energy minimization. These two scoring systems do not combine naturally. The earliest practical implementation of a simplified version of the Sankoff algorithm was Gorodkin's FOLDALIGN [34] which simply utilized an *ad hoc *additive combination of alignment scoring matrices and base pair maximization. It would be advantageous to find a more unified and formally justifiable scoring treatment.

Basing the overall objective function purely on thermodynamics seems problematic, because it is hard to see how to express inherently stochastic evolutionary events in terms of free energies. Nonetheless, Mathews and Turner's Dynalign program [30] does do this, and performs well: it uses a Sankoff algorithm to find an optimal alignment and consensus structure for a pair of RNA sequences by optimizing the sum of the two structures' predicted free energies, while using an *ad hoc *pseudoenergy penalty for indels.

Deriving a combined objective function in terms of probability theory seems more straightforward. One can find a consensus structure with maximum posterior probability by finding the structure that maximizes the joint probability of both the sequences and the structure. A fully probabilistic treatment of the simultaneous alignment and folding of two RNA sequences has been described using pairwise stochastic context-free grammars (pairSCFGs) and an algorithm essentially identical to the Sankoff algorithm [28,31].

Any practical implementation of the Sankoff algorithm must also find a way to reduce its prohibitive computational complexity. Gorodkin's original FOLDALIGN[34] did not permit multi-branch loops, focusing instead on the simpler problem of stem finding. Mathews and Turner's Dynalign implementation assumes a global alignment in order to fix a window of width ℳ
 MathType@MTEF@5@5@+=feaafiart1ev1aaatCvAUfKttLearuWrP9MDH5MBPbIqV92AaeXatLxBI9gBamrtHrhAL1wy0L2yHvtyaeHbnfgDOvwBHrxAJfwnaebbnrfifHhDYfgasaacH8akY=wiFfYdH8Gipec8Eeeu0xXdbba9frFj0=OqFfea0dXdd9vqai=hGuQ8kuc9pgc9s8qqaq=dirpe0xb9q8qiLsFr0=vr0=vr0dc8meaabaqaciaacaGaaeqabaWaaeGaeaaakeaaimaacqWFZestaaa@3790@ around the alignment diagonal [30,35,33]. In other words, a position in sequence **x **is restricted to align within ± ℳ
 MathType@MTEF@5@5@+=feaafiart1ev1aaatCvAUfKttLearuWrP9MDH5MBPbIqV92AaeXatLxBI9gBamrtHrhAL1wy0L2yHvtyaeHbnfgDOvwBHrxAJfwnaebbnrfifHhDYfgasaacH8akY=wiFfYdH8Gipec8Eeeu0xXdbba9frFj0=OqFfea0dXdd9vqai=hGuQ8kuc9pgc9s8qqaq=dirpe0xb9q8qiLsFr0=vr0=vr0dc8meaabaqaciaacaGaaeqabaWaaeGaeaaakeaaimaacqWFZestaaa@3790@ of the same position in sequence **y**. A recent update to FOLDALIGN uses a similar windowing approach, limiting both the alignment width and folding distance[32]. Hofacker precomputes base pairing probability matrices and then attempts to determine an alignment between these matrices[36]. Holmes uses an alternative approach, restricting a pairSCFG to searching for the structural alignment among a set of precomputed single sequence secondary structures and a set of precomputed alignments[28,31].

Here we describe a practical constrained pairwise global RNA structural alignment algorithm using pairSCFGs. We first describe a pairSCFG description of the structural alignment algorithm which extends our earlier work on SCFG design [37]. We parameterize and evaluate this full (unconstrained) structural alignment algorithm. We then outline a constrained structural algorithm which assumes knowledge of a few fixed positions, or "pins", within the alignment. We derive high-confidence pins from posterior probabilities in a probabilistic primary sequence alignment. We find that the constrained algorithm greatly improves CPU and memory requirements with minimal impact on alignment and structure prediction accuracy. Finally, we compare the performance of our algorithm with the other constrained RNA structural alignment implementations: Dynalign [30], Stemloc [31], FOLDALIGN [32], and PMcomp [36].

## Results and discussion

### Algorithms

Stochastic context-free grammars (SCFGs) are a probabilistic framework for modeling non-pseudoknotted secondary structure of RNAs. We assume familiarity with SCFGs as described in [38] and [39]. SCFGs provide a toolkit for designing RNA structure prediction and alignment methods. Many different SCFG designs are possible for describing an RNA structural alignment. A good design would be one that captures the informative statistics of RNA structural features – base pair stacking correlations, loop length preferences, and so on – with as much biological realism as possible. On the other hand, a good design must also be simple enough that it has a reasonable number of free parameters so it can be trained on known data.

Ideally the SCFG design should be formally *unambiguous *with respect to both secondary structure and alignment [40,38]. That is, an SCFG alignment algorithm will produce an optimal *parse tree π *that describes how the grammar aligns and scores the two sequences. There must be a strict one-to-one relationship between parse trees and alignments, as well as parse trees and base-paired secondary structures, in order for the optimal parse tree to be interpretable as an optimal alignment and structure.

We consider one alignment to be a set of aligned residue pairs. Any two alignments that yield the same set of aligned residue pairs are considered identical. That is,

a-bd

ef-g

and

ab-d

e-fg

are the same alignment (a,e),(d,g). A grammar is *alignment-ambiguous *if there exists an alignment that can be generated by more than one possible parse tree.

Similarly, we consider one secondary structure to be a set of base pairs. Any two structures that yield the same set of base pairs are the same structure. A grammar is *structurally ambiguous *if there exists a secondary structure that can be generated by more than one possible parse tree.

The consequence of using an ambiguous grammar is that the probability of a single alignment or structure may be spread across many parse trees that describe the same set of aligned residues or base pairs, therefore an optimal parse tree could represent a less optimal alignment or structure that merely has fewer alternative parse tree representations. Our previous work [38] indicated that this is a significant practical concern. Grammar ambiguity is not usually an issue for non-probabilistic scoring systems that simply seek to maximize an arbitrary score.

In our previous work [38] we demonstrated that small simple unambiguous stochastic context-free grammar designs can give reasonably good single sequence RNA structure prediction performance. In particular, a grammar such as:

G_*s *_    *S *→ *aS *| *T *| *ε*

*T *→ *Ta *| *aPa*' | *TaPa*'

*P *→ *aPa*' | *N*

*N *→ *aS *| *Ta *| *TaPa*'

was found to give good secondary structure performance for a reasonably small number of parameters. The notation *T *→ *aPa*' implies a basepairs with *a*', '|' denotes 'or' between production rules, and *ε *is the null string used to represent an ending production. The symbols *a *is used generically to represent any nucleotide of RNA (a terminal symbol). All 16 possible base pairs are permitted, including non-canonical pairs with low probability.

We can extend this grammar to the problem of pairwise structural alignment, the simultaneous sequence alignment and structure prediction of two sequences, simply by making it generate two correlated sequences instead of one. A different grammar from [38] ("*G*6") was found to give slightly better single sequence structure prediction performance, but appears to be difficult to extend to an alignment-unambiguous pair grammar.

To handle structural alignment, the SCFG is extended to emit a correlated pair of sequences, **x **and **y**. As we are interested in identifying shared structure, we first extend base pairing states to the pairwise case, abPa′b′
 MathType@MTEF@5@5@+=feaafiart1ev1aaatCvAUfKttLearuWrP9MDH5MBPbIqV92AaeXatLxBI9gBaebbnrfifHhDYfgasaacH8akY=wiFfYdH8Gipec8Eeeu0xXdbba9frFj0=OqFfea0dXdd9vqai=hGuQ8kuc9pgc9s8qqaq=dirpe0xb9q8qiLsFr0=vr0=vr0dc8meaabaqaciaacaGaaeqabaqabeGadaaakeaafaqabeGabaaabaGaemyyaegabaGaemOyaigaaiabdcfaqvaabeqaceaaaeaacuWGHbqygaqbaaqaaiqbdkgaIzaafaaaaaaa@3337@ where the notation implies *a *basepairs with *a*' in sequence **x **and *b *basepairs with *b*' in sequence **y**. This rule effectively captures basepair correlations observed in evolutionarily conserved secondary structures. In unpaired regions, the grammar reverts to an alignment algorithm so we logically replace each unpaired region of the SCFG with a typical pairHMM model of alignment[39,41]. The resulting pairwise SCFG (pairSCFG) grammar is:

S→abS|a−Lx|−bLy|T|εT→Tab|Rxa−|Ry−b|abPa′b′|TabPa′b′Lx→abS|a−Lx|T|εLy→abS|−bLy|T|εRx→Tab|Rxa−|abPa′b′|TabPa′b′Ry→Tab|Ry−b|abPa′b′|TabPa′b′P→abPa′b′|NN→abS|a−Lx|−bLy|Tab|Rxa−|Ry−b|TabPa′b′
 MathType@MTEF@5@5@+=feaafiart1ev1aaatCvAUfKttLearuWrP9MDH5MBPbIqV92AaeXatLxBI9gBaebbnrfifHhDYfgasaacH8akY=wiFfYdH8Gipec8Eeeu0xXdbba9frFj0=OqFfea0dXdd9vqai=hGuQ8kuc9pgc9s8qqaq=dirpe0xb9q8qiLsFr0=vr0=vr0dc8meaabaqaciaacaGaaeqabaqabeGadaaakqaabeqaaiabdofatjabgkziUwaabeqaceaaaeaacqWGHbqyaeaacqWGIbGyaaGaem4uamLaeiiFaWxbaeqabiqaaaqaaiabdggaHbqaaiabgkHiTaaacqWGmbatdaWgaaWcbaGaemiEaGhabeaakiabcYha8vaabiqaceaaaeaacqGHsislaeaacqWGIbGyaaGaemitaW0aaSbaaSqaaiabdMha5bqabaGccqGG8baFcqWGubavcqGG8baFiiGacqWF1oqzaeaacqWGubavcqGHsgIRcqWGubavfaqabeGabaaabaGaemyyaegabaGaemOyaigaaiabcYha8jabdkfasnaaBaaaleaacqWG4baEaeqaaOqbaeqabiqaaaqaaiabdggaHbqaaiabgkHiTaaacqGG8baFcqWGsbGudaWgaaWcbaGaemyEaKhabeaakuaabeqaceaaaeaacqGHsislaeaacqWGIbGyaaGaeiiFaWxbaeqabiqaaaqaaiabdggaHbqaaiabdkgaIbaacqWGqbaufaqabeGabaaabaGafmyyaeMbauaaaeaacuWGIbGygaqbaaaacqGG8baFcqWGubavfaqabeGabaaabaGaemyyaegabaGaemOyaigaaiabdcfaqvaabeqaceaaaeaacuWGHbqygaqbaaqaaiqbdkgaIzaafaaaaaqaaiabdYeamnaaBaaaleaacqWG4baEaeqaaOGaeyOKH4AbaeqabiqaaaqaaiabdggaHbqaaiabdkgaIbaacqWGtbWucqGG8baFfaqabeGabaaabaGaemyyaegabaGaeyOeI0caaiabdYeamnaaBaaaleaacqWG4baEaeqaaOGaeiiFaWNaemivaqLaeiiFaWNae8xTdugabaGaemitaW0aaSbaaSqaaiabdMha5bqabaGccqGHsgIRfaqabeGabaaabaGaemyyaegabaGaemOyaigaaiabdofatjabcYha8vaabeqaceaaaeaacqGHsislaeaacqWGIbGyaaGaemitaW0aaSbaaSqaaiabdMha5bqabaGccqGG8baFcqWGubavcqGG8baFcqWF1oqzaeaacqWGsbGudaWgaaWcbaGaemiEaGhabeaakiabgkziUkabdsfauvaabeqaceaaaeaacqWGHbqyaeaacqWGIbGyaaGaeiiFaWNaemOuai1aaSbaaSqaaiabdIha4bqabaGcfaqabeGabaaabaGaemyyaegabaGaeyOeI0caaiabcYha8vaabeqaceaaaeaacqWGHbqyaeaacqWGIbGyaaGaemiuaavbaeqabiqaaaqaaiqbdggaHzaafaaabaGafmOyaiMbauaaaaGaeiiFaWNaemivaqvbaeqabiqaaaqaaiabdggaHbqaaiabdkgaIbaacqWGqbaufaqabeGabaaabaGafmyyaeMbauaaaeaacuWGIbGygaqbaaaaaeaacqWGsbGudaWgaaWcbaGaemyEaKhabeaakiabgkziUkabdsfauvaabeqaceaaaeaacqWGHbqyaeaacqWGIbGyaaGaeiiFaWNaemOuai1aaSbaaSqaaiabdMha5bqabaGcfaqabeGabaaabaGaeyOeI0cabaGaemOyaigaaiabcYha8vaabeqaceaaaeaacqWGHbqyaeaacqWGIbGyaaGaemiuaavbaeqabiqaaaqaaiqbdggaHzaafaaabaGafmOyaiMbauaaaaGaeiiFaWNaemivaqvbaeqabiqaaaqaaiabdggaHbqaaiabdkgaIbaacqWGqbaufaqabeGabaaabaGafmyyaeMbauaaaeaacuWGIbGygaqbaaaaaeaacqWGqbaucqGHsgIRfaqabeGabaaabaGaemyyaegabaGaemOyaigaaiabdcfaqvaabeqaceaaaeaacuWGHbqygaqbaaqaaiqbdkgaIzaafaaaaiabcYha8jabd6eaobqaaiabd6eaojabgkziUwaabeqaceaaaeaacqWGHbqyaeaacqWGIbGyaaGaem4uamLaeiiFaWxbaeqabiqaaaqaaiabdggaHbqaaiabgkHiTaaacqWGmbatdaWgaaWcbaGaemiEaGhabeaakiabcYha8vaabeqaceaaaeaacqGHsislaeaacqWGIbGyaaGaemitaW0aaSbaaSqaaiabdMha5bqabaGccqGG8baFcqWGubavfaqabeGabaaabaGaemyyaegabaGaemOyaigaaiabcYha8jabdkfasnaaBaaaleaacqWG4baEaeqaaOqbaeqabiqaaaqaaiabdggaHbqaaiabgkHiTaaacqGG8baFcqWGsbGudaWgaaWcbaGaemyEaKhabeaakuaabeqaceaaaeaacqGHsislaeaacqWGIbGyaaGaeiiFaWNaemivaqvbaeqabiqaaaqaaiabdggaHbqaaiabdkgaIbaacqWGqbaufaqabeGabaaabaGafmyyaeMbauaaaeaacuWGIbGygaqbaaaaaaaa@1376@

which has eight nonterminals and 35 production rules. We postulate that this grammar is both structurally unambiguous and alignment-unambiguous (evidence is given in the Appendix).

This grammar lacks at least two features that are thought to be biologically important in RNA folding. It does not have stacking rules; base pair emissions are statistically independent of each other. It also does not have explicit length distributions for hairpin loops, bulge loops, interior loops, and multiloops. All length distributions are modeled in this grammar by rules of the general form *S *→ *aS*, which imply a geometric length distribution. Unambiguous SCFG designs that capture a more biologically realistic model of RNA are more complex, and we have deferred them to future work.

#### Maximum likelihood structure prediction

Given a parameterized pairSCFG model and two input sequences, we find the maximum likelihood parse tree (simultaneously the secondary structure and alignment) by a pairSCFG CYK algorithm [31]. More formally, given a SCFG and the parameters of the model (Θ) the Cocke-Younger-Kasami (CYK) algorithm finds the optimal parse tree π^
 MathType@MTEF@5@5@+=feaafiart1ev1aaatCvAUfKttLearuWrP9MDH5MBPbIqV92AaeXatLxBI9gBaebbnrfifHhDYfgasaacH8akY=wiFfYdH8Gipec8Eeeu0xXdbba9frFj0=OqFfea0dXdd9vqai=hGuQ8kuc9pgc9s8qqaq=dirpe0xb9q8qiLsFr0=vr0=vr0dc8meaabaqaciaacaGaaeqabaqabeGadaaakeaaiiGacuWFapaCgaqcaaaa@2E80@

π^
 MathType@MTEF@5@5@+=feaafiart1ev1aaatCvAUfKttLearuWrP9MDH5MBPbIqV92AaeXatLxBI9gBaebbnrfifHhDYfgasaacH8akY=wiFfYdH8Gipec8Eeeu0xXdbba9frFj0=OqFfea0dXdd9vqai=hGuQ8kuc9pgc9s8qqaq=dirpe0xb9q8qiLsFr0=vr0=vr0dc8meaabaqaciaacaGaaeqabaqabeGadaaakeaaiiGacuWFapaCgaqcaaaa@2E80@ = argmax_*π *_*P*(*π*, **X**|G
 MathType@MTEF@5@5@+=feaafiart1ev1aaatCvAUfKttLearuWrP9MDH5MBPbIqV92AaeXatLxBI9gBamrtHrhAL1wy0L2yHvtyaeHbnfgDOvwBHrxAJfwnaebbnrfifHhDYfgasaacH8akY=wiFfYdH8Gipec8Eeeu0xXdbba9frFj0=OqFfea0dXdd9vqai=hGuQ8kuc9pgc9s8qqaq=dirpe0xb9q8qiLsFr0=vr0=vr0dc8meaabaqaciaacaGaaeqabaWaaeGaeaaakeaaimaacqWFge=raaa@382D@, Θ)

for the set of input sequences **X**. For single sequence SCFGs, **X **= {**x**} whereas for a pairSCFG **X **= {**x**, **y**}. This dynamic programming algorithm works by calculating the likelihood of all partial subsequences of the inputs, starting with zero length sequences and working outward to their full lengths. In general each nonterminal of a grammar requires an additional dynamic programming matrix. For example, the *P *nonterminal of G_*s*_, described by production rules *P *→ *aPa*' | *N *is computed as:

P(i,j)=max⁡{P(i+1,j−1)+log⁡p(P→xiPxj)P(i,j)+log⁡p(P→N)
 MathType@MTEF@5@5@+=feaafiart1ev1aaatCvAUfKttLearuWrP9MDH5MBPbIqV92AaeXatLxBI9gBaebbnrfifHhDYfgasaacH8akY=wiFfYdH8Gipec8Eeeu0xXdbba9frFj0=OqFfea0dXdd9vqai=hGuQ8kuc9pgc9s8qqaq=dirpe0xb9q8qiLsFr0=vr0=vr0dc8meaabaqaciaacaGaaeqabaqabeGadaaakeaacqWGqbaucqGGOaakcqWGPbqAcqGGSaalcqWGQbGAcqGGPaqkcqGH9aqpcyGGTbqBcqGGHbqycqGG4baEdaGabeqaauaabaqaceaaaeaacqWGqbaucqGGOaakcqWGPbqAcqGHRaWkcqaIXaqmcqGGSaalcqWGQbGAcqGHsislcqaIXaqmcqGGPaqkcqGHRaWkcyGGSbaBcqGGVbWBcqGGNbWzcqWGWbaCcqGGOaakcqWGqbaucqGHsgIRcqWG4baEdaWgaaWcbaGaemyAaKgabeaakiabdcfaqjabdIha4naaBaaaleaacqWGQbGAaeqaaOGaeiykaKcabaGaemiuaaLaeiikaGIaemyAaKMaeiilaWIaemOAaOMaeiykaKIaey4kaSIagiiBaWMaei4Ba8Maei4zaCMaemiCaaNaeiikaGIaemiuaaLaeyOKH4QaemOta4KaeiykaKcaaaGaay5Eaaaaaa@68CD@

Whereas in the pairSCFG the *P *nonterminal, described by production rules *P *→ abPa′b′
 MathType@MTEF@5@5@+=feaafiart1ev1aaatCvAUfKttLearuWrP9MDH5MBPbIqV92AaeXatLxBI9gBaebbnrfifHhDYfgasaacH8akY=wiFfYdH8Gipec8Eeeu0xXdbba9frFj0=OqFfea0dXdd9vqai=hGuQ8kuc9pgc9s8qqaq=dirpe0xb9q8qiLsFr0=vr0=vr0dc8meaabaqaciaacaGaaeqabaqabeGadaaakeaafaqabeGabaaabaGaemyyaegabaGaemOyaigaaiabdcfaqvaabeqaceaaaeaacuWGHbqygaqbaaqaaiqbdkgaIzaafaaaaaaa@3337@ | *N*, is computed as:

P(i,j;k,l)=max⁡{P(i+1,j−1;k+1,l−1)+log⁡p(P→xiyiPxjyj)N(i,j;k,l)+log⁡p(P→N)
 MathType@MTEF@5@5@+=feaafiart1ev1aaatCvAUfKttLearuWrP9MDH5MBPbIqV92AaeXatLxBI9gBaebbnrfifHhDYfgasaacH8akY=wiFfYdH8Gipec8Eeeu0xXdbba9frFj0=OqFfea0dXdd9vqai=hGuQ8kuc9pgc9s8qqaq=dirpe0xb9q8qiLsFr0=vr0=vr0dc8meaabaqaciaacaGaaeqabaqabeGadaaakeaacqWGqbaucqGGOaakcqWGPbqAcqGGSaalcqWGQbGAcqGG7aWocqWGRbWAcqGGSaalcqWGSbaBcqGGPaqkcqGH9aqpcyGGTbqBcqGGHbqycqGG4baEdaGabeqaauaabaqaceaaaeaacqWGqbaucqGGOaakcqWGPbqAcqGHRaWkcqaIXaqmcqGGSaalcqWGQbGAcqGHsislcqaIXaqmcqGG7aWocqWGRbWAcqGHRaWkcqaIXaqmcqGGSaalcqWGSbaBcqGHsislcqaIXaqmcqGGPaqkcqGHRaWkcyGGSbaBcqGGVbWBcqGGNbWzcqWGWbaCcqGGOaakcqWGqbaucqGHsgIRfaqabeGabaaabaGaemiEaG3aaSbaaSqaaiabdMgaPbqabaaakeaacqWG5bqEdaWgaaWcbaGaemyAaKgabeaaaaGccqWGqbaufaqabeGabaaabaGaemiEaG3aaSbaaSqaaiabdQgaQbqabaaakeaacqWG5bqEdaWgaaWcbaGaemOAaOgabeaaaaGccqGGPaqkaeaacqWGobGtcqGGOaakcqWGPbqAcqGGSaalcqWGQbGAcqGG7aWocqWGRbWAcqGGSaalcqWGSbaBcqGGPaqkcqGHRaWkcyGGSbaBcqGGVbWBcqGGNbWzcqWGWbaCcqGGOaakcqWGqbaucqGHsgIRcqWGobGtcqGGPaqkaaaacaGL7baaaaa@8086@

**Figure 1:**
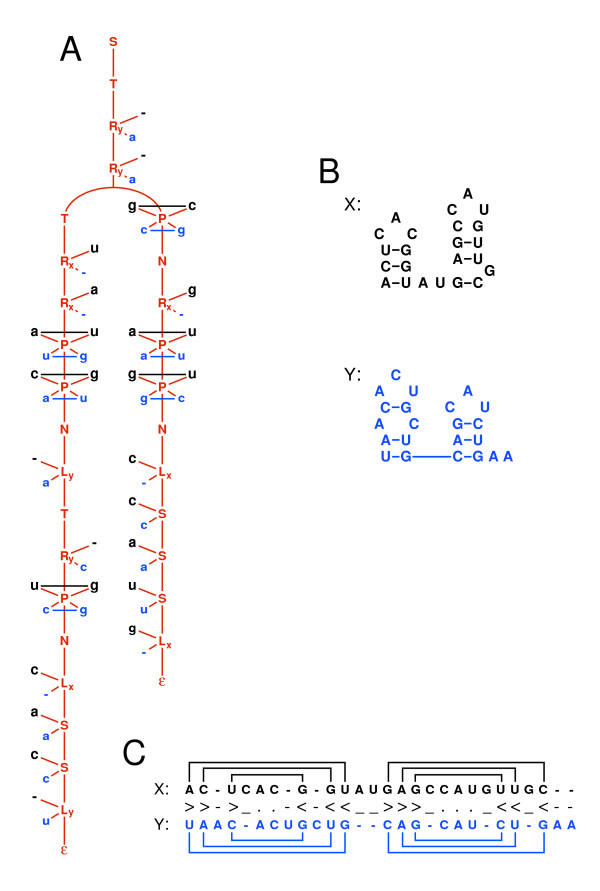
**Example parse tree for pairSCFG**. *Panel A *shows an example parse tree for the pairSCFG described in the text. The grammar emits two correlated sequences, **x **(above, in black) and **y **(below, in blue). The individual structures are shown in *Panel B*. The resulting structural alignment is shown in *Panel C*, with lines connecting base pairs in each sequence. The annotation string is shown between sequence **x **and **y **(see Appendix for details). Note that the structure for **x **(above, in black) has what is most likely an unconserved base pair **C**·**G **in the second stem of the structure.

From this example, the correspondence between grammar rules and the CYK algorithm should be clear. An example parse tree from the pairSCFG is shown in Figure 1. This algorithm is O(N^2^M^2^) in memory and O(N^3^M^3^) in time for two sequences of lengths N and M. As we are focusing on the global alignment of homologous RNAs, we assume that the lengths of the two sequences are roughly comparable and the algorithm is O(N^4^) in memory and O(N^6^) in time.

#### Parameterization

Given the set of pairSCFG production rules above, we need to determine all the necessary probability parameters. As is common for many stochastic models [39,31], we distinguish *transition *parameters for the probability of using a production rule from *emission *parameters that generate any nucleotide(s) from the rule.

We use two types of parameter tying to reduce the number of free parameters in the model. First, we assume that the model should be symmetric, assigning the same probability to **x**, **y **and **y**, **x**. Second, we tie several additional "equivalent" parameters together, as follows.

An untied version of the pairSCFG would require 2234 emission parameters: there are 12 rules that generate single nucleotides (4 parameters apiece), 8 aligned nucleotide rules (16 parameters apiece), and 8 aligned base pair nucleotide rules (256 parameters apiece). We reduce these to one emission distribution for single (unaligned, unpaired) nucleotides, one symmetric 4 × 4 matrix for emitting unpaired aligned residues, and one symmetric 16 × 16 matrix for aligned base pairs. The tied model has 150 emission parameters, of which 129 are free.

The untied pairSCFG has 35 production rules, hence 35 transition probabilities. We group transition parameters such that gaps are treated equivalently in **x **and **y **by tying together the sets of parameters utilized for "gap open", "gap extend", and "gap closure". For example S→a−Lx
 MathType@MTEF@5@5@+=feaafiart1ev1aaatCvAUfKttLearuWrP9MDH5MBPbIqV92AaeXatLxBI9gBaebbnrfifHhDYfgasaacH8akY=wiFfYdH8Gipec8Eeeu0xXdbba9frFj0=OqFfea0dXdd9vqai=hGuQ8kuc9pgc9s8qqaq=dirpe0xb9q8qiLsFr0=vr0=vr0dc8meaabaqaciaacaGaaeqabaqabeGadaaakeaacqWGtbWucqGHsgIRfaqabeGabaaabaGaemyyaegabaGaeyOeI0caaiabdYeamnaaBaaaleaacqWG4baEaeqaaaaa@34D3@ must equal S→−bLy
 MathType@MTEF@5@5@+=feaafiart1ev1aaatCvAUfKttLearuWrP9MDH5MBPbIqV92AaeXatLxBI9gBaebbnrfifHhDYfgasaacH8akY=wiFfYdH8Gipec8Eeeu0xXdbba9frFj0=OqFfea0dXdd9vqai=hGuQ8kuc9pgc9s8qqaq=dirpe0xb9q8qiLsFr0=vr0=vr0dc8meaabaqaciaacaGaaeqabaqabeGadaaakeaacqWGtbWucqGHsgIRfaqabeGabaaabaGaeyOeI0cabaGaemOyaigaaiabdYeamnaaBaaaleaacqWG5bqEaeqaaaaa@34D7@ as both rules are used as "gap open" parameters. Likewise, the rules of *L*_*x *_are tied to the rules of *L*_*y *_as they both represent "gap extend" and "gap closure" rules. The tied model has 22 transition parameters, of which 16 are free.

The 172 parameters of the pairSCFG were then estimated from the frequencies observed in annotated ribosomal RNA secondary structures from multiple alignments in the European Ribosomal Database [42,43]. Sequences containing more than 5% ambiguous bases or with less than 40% base pairing are discarded. The resulting data set was then filtered to remove sequences with greater than 80% idenity to avoid overcounting nearly identical alignments. The two resulting multiple sequence alignments contain 707 sequences, of which 568 are small subunit and 139 are large subunit, and a total of 1,233,293 nucleotides. The parse tree for each implicit pairwise structural alignment is determined, and the number of occurrences of each production type is counted. All pairs, excluding self-to-self comparisons, are counted. Probabilities are estimated from the counts using a Laplace (plus-one) prior [39].

Because the training dataset consisted of many sequences but only two different RNA structures (large and small subunit ribosomal RNA), we had some concern that the model might fail to generalize to other, shorter, structural RNAs. To address this concern, we compared two models trained on two independent training sets. The first is the aforementioned rRNA dataset. The second is composed of all seed Rfam v7.0 families marked as published. Each family was filtered at 80% identity and those with fewer than two sequences remaining were subsequently removed. The SSU Rfam family was removed to avoid overlap with the rRNA training set. The resulting dataset consisted of 96 families, a total of 2054 sequences with an average length of 137 nucleotides, and a total of 302,993 nucleotides.

**Figure 2:**
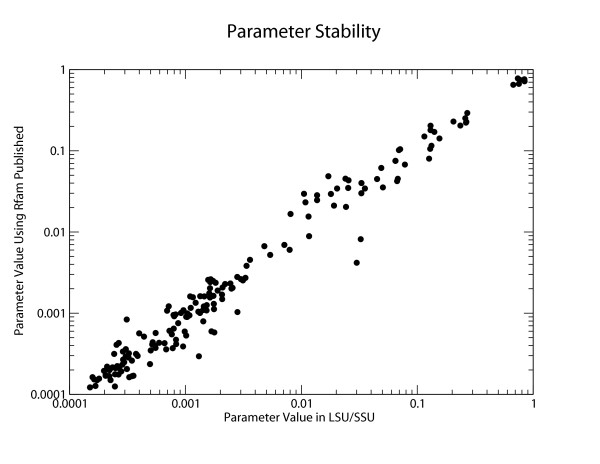
**Comparison of parameters using two different training datasets**. The parameter values of the LSU/SSU trained model are plotted on a log scale against the parameters determined using the published Rfam training set. The Rfam training set excludes the SSU family to avoid overlap between the training sets. Because the plot is roughly linear (with a slope of 1), the parameter values appear to be relatively robust with respect to the source of the training data. Interestingly, the two off diagonal elements refer to the probabilities that a GU or UG pairs remain unchanged across the alignment (ie. a GU pair aligns to a GU pair), which are higher in the ribosomal dataset.

Figure 2 shows a scatter plot comparing the 172 parameter values under the two training protocols. Differences appear to be minor, so the rRNA-trained model does not appear to be obviously overfitted.

#### Constrained CYK algorithm

At *O*(*N*^4^) memory and *O*(*N*^6^) time, the full (unconstrained) pairSCFG CYK algorithm is barely practical. On current computers, it becomes unreasonable for RNAs of more than about 100 nucleotides in length. Our heuristic strategy is to constrain the algorithm by a small number of primary sequence alignment *pins *representing confidently aligned pairs of residues. Using pins (or anchors) to fix portions of the alignment is used by a number of sequence alignment programs to allow very long alignments [44]. Given a set of pins (from whatever source), the constrained pairSCFG CYK algorithm is as follows:

By convention, *i*, *a*, and *j *are indices in **x **and *k*, *b*, and *l *are indices in **y**. Sequence **x **is length *M *and **y **is length *N*. The subsequence *i*...*j *aligns to *k*...*l*. For each nonterminal in the grammar we keep a four dimensional matrix, *i*, *j*, *k*, *l*. The indices *a *and *b *locate the split points in a bifurcation rule. With bifurcation rules, we must consider all possible split points, therefore 1 ≤ *i *<*a *<*j *≤ *M *and 1 ≤ *k *<*b *<*l *≤ *N*.

We define a pin *q*_*z *_as a coordinate pair (*q*_*z *_(*x*), *q*_*z *_(*y*)) where the nucleotide *q*_*z *_(*x*) aligns to the nucleotide *q*_*z *_(*y*). The ordered set of alignment pins Q
 MathType@MTEF@5@5@+=feaafiart1ev1aaatCvAUfKttLearuWrP9MDH5MBPbIqV92AaeXatLxBI9gBamrtHrhAL1wy0L2yHvtyaeHbnfgDOvwBHrxAJfwnaebbnrfifHhDYfgasaacH8akY=wiFfYdH8Gipec8Eeeu0xXdbba9frFj0=OqFfea0dXdd9vqai=hGuQ8kuc9pgc9s8qqaq=dirpe0xb9q8qiLsFr0=vr0=vr0dc8meaabaqaciaacaGaaeqabaWaaeGaeaaakeaaimaacqWFqeFuaaa@3841@ contains *Z *pins, numbered from the 5' end of sequence **x**, i.e. *z *= 1..*Z*. We always define two pins (*Z *≥ 2) as boundary conditions: one which comes before the 5' nucleotide of each sequence *q*_1 _= (0, 0) and one which follows the 3' nucleotide of each sequence *q*_*z *_= (*M *+ 1, *N *+ 1).

We seek to calculate

π^
 MathType@MTEF@5@5@+=feaafiart1ev1aaatCvAUfKttLearuWrP9MDH5MBPbIqV92AaeXatLxBI9gBaebbnrfifHhDYfgasaacH8akY=wiFfYdH8Gipec8Eeeu0xXdbba9frFj0=OqFfea0dXdd9vqai=hGuQ8kuc9pgc9s8qqaq=dirpe0xb9q8qiLsFr0=vr0=vr0dc8meaabaqaciaacaGaaeqabaqabeGadaaakeaaiiGacuWFapaCgaqcaaaa@2E80@ = argmax_*π *_*P *(*π*, **x**, **y **| Q
 MathType@MTEF@5@5@+=feaafiart1ev1aaatCvAUfKttLearuWrP9MDH5MBPbIqV92AaeXatLxBI9gBamrtHrhAL1wy0L2yHvtyaeHbnfgDOvwBHrxAJfwnaebbnrfifHhDYfgasaacH8akY=wiFfYdH8Gipec8Eeeu0xXdbba9frFj0=OqFfea0dXdd9vqai=hGuQ8kuc9pgc9s8qqaq=dirpe0xb9q8qiLsFr0=vr0=vr0dc8meaabaqaciaacaGaaeqabaWaaeGaeaaakeaaimaacqWFqeFuaaa@3841@, G
 MathType@MTEF@5@5@+=feaafiart1ev1aaatCvAUfKttLearuWrP9MDH5MBPbIqV92AaeXatLxBI9gBamrtHrhAL1wy0L2yHvtyaeHbnfgDOvwBHrxAJfwnaebbnrfifHhDYfgasaacH8akY=wiFfYdH8Gipec8Eeeu0xXdbba9frFj0=OqFfea0dXdd9vqai=hGuQ8kuc9pgc9s8qqaq=dirpe0xb9q8qiLsFr0=vr0=vr0dc8meaabaqaciaacaGaaeqabaWaaeGaeaaakeaaimaacqWFge=raaa@382D@, Θ)

the highest probability parse tree π^
 MathType@MTEF@5@5@+=feaafiart1ev1aaatCvAUfKttLearuWrP9MDH5MBPbIqV92AaeXatLxBI9gBaebbnrfifHhDYfgasaacH8akY=wiFfYdH8Gipec8Eeeu0xXdbba9frFj0=OqFfea0dXdd9vqai=hGuQ8kuc9pgc9s8qqaq=dirpe0xb9q8qiLsFr0=vr0=vr0dc8meaabaqaciaacaGaaeqabaqabeGadaaakeaaiiGacuWFapaCgaqcaaaa@2E80@ for the sequences **x **and **y **given the set of alignment pins Q
 MathType@MTEF@5@5@+=feaafiart1ev1aaatCvAUfKttLearuWrP9MDH5MBPbIqV92AaeXatLxBI9gBamrtHrhAL1wy0L2yHvtyaeHbnfgDOvwBHrxAJfwnaebbnrfifHhDYfgasaacH8akY=wiFfYdH8Gipec8Eeeu0xXdbba9frFj0=OqFfea0dXdd9vqai=hGuQ8kuc9pgc9s8qqaq=dirpe0xb9q8qiLsFr0=vr0=vr0dc8meaabaqaciaacaGaaeqabaWaaeGaeaaakeaaimaacqWFqeFuaaa@3841@, the pairwise grammar G
 MathType@MTEF@5@5@+=feaafiart1ev1aaatCvAUfKttLearuWrP9MDH5MBPbIqV92AaeXatLxBI9gBamrtHrhAL1wy0L2yHvtyaeHbnfgDOvwBHrxAJfwnaebbnrfifHhDYfgasaacH8akY=wiFfYdH8Gipec8Eeeu0xXdbba9frFj0=OqFfea0dXdd9vqai=hGuQ8kuc9pgc9s8qqaq=dirpe0xb9q8qiLsFr0=vr0=vr0dc8meaabaqaciaacaGaaeqabaWaaeGaeaaakeaaimaacqWFge=raaa@382D@, and the parameters of the model Θ. If the pins are "correct" relative to the unconstrained structural alignment, i.e. (∀ *z *∈ Q
 MathType@MTEF@5@5@+=feaafiart1ev1aaatCvAUfKttLearuWrP9MDH5MBPbIqV92AaeXatLxBI9gBamrtHrhAL1wy0L2yHvtyaeHbnfgDOvwBHrxAJfwnaebbnrfifHhDYfgasaacH8akY=wiFfYdH8Gipec8Eeeu0xXdbba9frFj0=OqFfea0dXdd9vqai=hGuQ8kuc9pgc9s8qqaq=dirpe0xb9q8qiLsFr0=vr0=vr0dc8meaabaqaciaacaGaaeqabaWaaeGaeaaakeaaimaacqWFqeFuaaa@3841@ : {*q*_*z *_(*x*), *q*_*z *_(*y*)}) ∈ π^
 MathType@MTEF@5@5@+=feaafiart1ev1aaatCvAUfKttLearuWrP9MDH5MBPbIqV92AaeXatLxBI9gBaebbnrfifHhDYfgasaacH8akY=wiFfYdH8Gipec8Eeeu0xXdbba9frFj0=OqFfea0dXdd9vqai=hGuQ8kuc9pgc9s8qqaq=dirpe0xb9q8qiLsFr0=vr0=vr0dc8meaabaqaciaacaGaaeqabaqabeGadaaakeaaiiGacuWFapaCgaqcaaaa@2E80@ = argmax_*π *_*P *(*π*, **x**, **y **| G
 MathType@MTEF@5@5@+=feaafiart1ev1aaatCvAUfKttLearuWrP9MDH5MBPbIqV92AaeXatLxBI9gBamrtHrhAL1wy0L2yHvtyaeHbnfgDOvwBHrxAJfwnaebbnrfifHhDYfgasaacH8akY=wiFfYdH8Gipec8Eeeu0xXdbba9frFj0=OqFfea0dXdd9vqai=hGuQ8kuc9pgc9s8qqaq=dirpe0xb9q8qiLsFr0=vr0=vr0dc8meaabaqaciaacaGaaeqabaWaaeGaeaaakeaaimaacqWFge=raaa@382D@, Θ), then the constrained algorithm is guaranteed to find the same optimal structural alignment π^
 MathType@MTEF@5@5@+=feaafiart1ev1aaatCvAUfKttLearuWrP9MDH5MBPbIqV92AaeXatLxBI9gBaebbnrfifHhDYfgasaacH8akY=wiFfYdH8Gipec8Eeeu0xXdbba9frFj0=OqFfea0dXdd9vqai=hGuQ8kuc9pgc9s8qqaq=dirpe0xb9q8qiLsFr0=vr0=vr0dc8meaabaqaciaacaGaaeqabaqabeGadaaakeaaiiGacuWFapaCgaqcaaaa@2E80@ as generated by the unconstrained algorithm.

**Figure 3:**
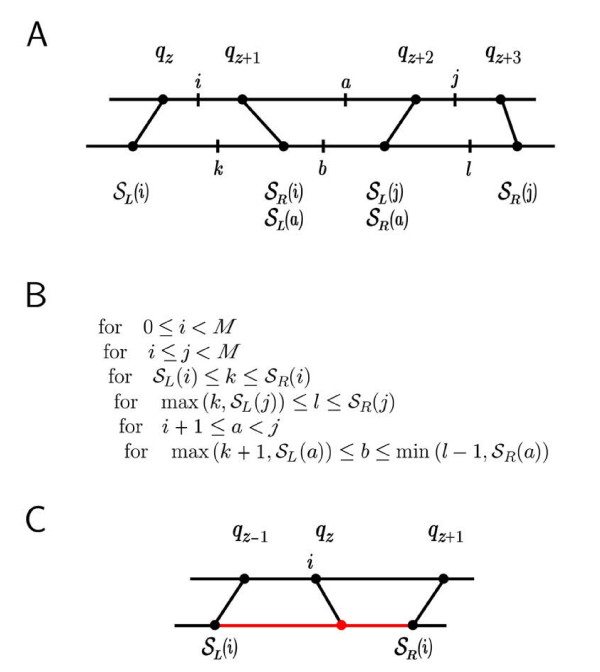
**Constrained Sankoff Algorithm**. *Panel A: *A cartoon depicting an example problem with four pins shown as dumbbells, labeled *q*_*z *_to *q*_*z*+3_, which connect the two sequences. The indices *i*, *a*, and *j *are positions in the first sequence **x**; *k*, *b*, and *l *are positions in the second sequence **y**. The corresponding segment edges S
 MathType@MTEF@5@5@+=feaafiart1ev1aaatCvAUfKttLearuWrP9MDH5MBPbIqV92AaeXatLxBI9gBamrtHrhAL1wy0L2yHvtyaeHbnfgDOvwBHrxAJfwnaebbnrfifHhDYfgasaacH8akY=wiFfYdH8Gipec8Eeeu0xXdbba9frFj0=OqFfea0dXdd9vqai=hGuQ8kuc9pgc9s8qqaq=dirpe0xb9q8qiLsFr0=vr0=vr0dc8meaabaqaciaacaGaaeqabaWaaeGaeaaakeaaimaacqWFse=uaaa@3845@_*L *_and S
 MathType@MTEF@5@5@+=feaafiart1ev1aaatCvAUfKttLearuWrP9MDH5MBPbIqV92AaeXatLxBI9gBamrtHrhAL1wy0L2yHvtyaeHbnfgDOvwBHrxAJfwnaebbnrfifHhDYfgasaacH8akY=wiFfYdH8Gipec8Eeeu0xXdbba9frFj0=OqFfea0dXdd9vqai=hGuQ8kuc9pgc9s8qqaq=dirpe0xb9q8qiLsFr0=vr0=vr0dc8meaabaqaciaacaGaaeqabaWaaeGaeaaakeaaimaacqWFse=uaaa@3845@_*R *_for each position in **x **are labeled. In this notation, *j *is the potential base pairing partner of *i*, *l *is the potential base pairing partner of *k*, and the subsequence *i*...*j *aligns with *k*...*l*. The indices *a *and *b *are the required for identifying bifurcation points. *Panel B: *The constrained structural alignment algorithm in pseudocode, where *M *is the length of sequence **x**, S
 MathType@MTEF@5@5@+=feaafiart1ev1aaatCvAUfKttLearuWrP9MDH5MBPbIqV92AaeXatLxBI9gBamrtHrhAL1wy0L2yHvtyaeHbnfgDOvwBHrxAJfwnaebbnrfifHhDYfgasaacH8akY=wiFfYdH8Gipec8Eeeu0xXdbba9frFj0=OqFfea0dXdd9vqai=hGuQ8kuc9pgc9s8qqaq=dirpe0xb9q8qiLsFr0=vr0=vr0dc8meaabaqaciaacaGaaeqabaWaaeGaeaaakeaaimaacqWFse=uaaa@3845@_*L *_(*i*) is the left edge of the segment containing the position *i*, and S
 MathType@MTEF@5@5@+=feaafiart1ev1aaatCvAUfKttLearuWrP9MDH5MBPbIqV92AaeXatLxBI9gBamrtHrhAL1wy0L2yHvtyaeHbnfgDOvwBHrxAJfwnaebbnrfifHhDYfgasaacH8akY=wiFfYdH8Gipec8Eeeu0xXdbba9frFj0=OqFfea0dXdd9vqai=hGuQ8kuc9pgc9s8qqaq=dirpe0xb9q8qiLsFr0=vr0=vr0dc8meaabaqaciaacaGaaeqabaWaaeGaeaaakeaaimaacqWFse=uaaa@3845@_*R*_(*i*) is the right edge of the segment containing *i*. The max in the range for *l *is required to handle the case where *i *and *j *share the same segment. The *b *range must be handled similarly. *Panel C: *The special case where the position under consideration is equivalent to a pin. In this case, we know the location of its alignment partner but must also consider the possibility of insertions in **y **which may occur before or after this pin.

Given Q
 MathType@MTEF@5@5@+=feaafiart1ev1aaatCvAUfKttLearuWrP9MDH5MBPbIqV92AaeXatLxBI9gBamrtHrhAL1wy0L2yHvtyaeHbnfgDOvwBHrxAJfwnaebbnrfifHhDYfgasaacH8akY=wiFfYdH8Gipec8Eeeu0xXdbba9frFj0=OqFfea0dXdd9vqai=hGuQ8kuc9pgc9s8qqaq=dirpe0xb9q8qiLsFr0=vr0=vr0dc8meaabaqaciaacaGaaeqabaWaaeGaeaaakeaaimaacqWFqeFuaaa@3841@, we define a segment S
 MathType@MTEF@5@5@+=feaafiart1ev1aaatCvAUfKttLearuWrP9MDH5MBPbIqV92AaeXatLxBI9gBamrtHrhAL1wy0L2yHvtyaeHbnfgDOvwBHrxAJfwnaebbnrfifHhDYfgasaacH8akY=wiFfYdH8Gipec8Eeeu0xXdbba9frFj0=OqFfea0dXdd9vqai=hGuQ8kuc9pgc9s8qqaq=dirpe0xb9q8qiLsFr0=vr0=vr0dc8meaabaqaciaacaGaaeqabaWaaeGaeaaakeaaimaacqWFse=uaaa@3845@(*i*) as the range of index *k *in **y **which must be considered for a particular *i *in **x**. A position *i *between pins *q*_*z *_and *q*_*z*+1 _has a segment S
 MathType@MTEF@5@5@+=feaafiart1ev1aaatCvAUfKttLearuWrP9MDH5MBPbIqV92AaeXatLxBI9gBamrtHrhAL1wy0L2yHvtyaeHbnfgDOvwBHrxAJfwnaebbnrfifHhDYfgasaacH8akY=wiFfYdH8Gipec8Eeeu0xXdbba9frFj0=OqFfea0dXdd9vqai=hGuQ8kuc9pgc9s8qqaq=dirpe0xb9q8qiLsFr0=vr0=vr0dc8meaabaqaciaacaGaaeqabaWaaeGaeaaakeaaimaacqWFse=uaaa@3845@(*i*) which implies *q*_*z *_(*y*) <*k *<*q*_*z*+1 _(*y*). We refer to edges of the range, in this case *q*_*z *_(*y*) and *q*_*z*+1 _(*y*) as S
 MathType@MTEF@5@5@+=feaafiart1ev1aaatCvAUfKttLearuWrP9MDH5MBPbIqV92AaeXatLxBI9gBamrtHrhAL1wy0L2yHvtyaeHbnfgDOvwBHrxAJfwnaebbnrfifHhDYfgasaacH8akY=wiFfYdH8Gipec8Eeeu0xXdbba9frFj0=OqFfea0dXdd9vqai=hGuQ8kuc9pgc9s8qqaq=dirpe0xb9q8qiLsFr0=vr0=vr0dc8meaabaqaciaacaGaaeqabaWaaeGaeaaakeaaimaacqWFse=uaaa@3845@_*L *_(*i*) and S
 MathType@MTEF@5@5@+=feaafiart1ev1aaatCvAUfKttLearuWrP9MDH5MBPbIqV92AaeXatLxBI9gBamrtHrhAL1wy0L2yHvtyaeHbnfgDOvwBHrxAJfwnaebbnrfifHhDYfgasaacH8akY=wiFfYdH8Gipec8Eeeu0xXdbba9frFj0=OqFfea0dXdd9vqai=hGuQ8kuc9pgc9s8qqaq=dirpe0xb9q8qiLsFr0=vr0=vr0dc8meaabaqaciaacaGaaeqabaWaaeGaeaaakeaaimaacqWFse=uaaa@3845@_*R *_(*i*) respectively. Refer to Figure 3 panel A for a labeled example. In the absence of constraints, Q
 MathType@MTEF@5@5@+=feaafiart1ev1aaatCvAUfKttLearuWrP9MDH5MBPbIqV92AaeXatLxBI9gBamrtHrhAL1wy0L2yHvtyaeHbnfgDOvwBHrxAJfwnaebbnrfifHhDYfgasaacH8akY=wiFfYdH8Gipec8Eeeu0xXdbba9frFj0=OqFfea0dXdd9vqai=hGuQ8kuc9pgc9s8qqaq=dirpe0xb9q8qiLsFr0=vr0=vr0dc8meaabaqaciaacaGaaeqabaWaaeGaeaaakeaaimaacqWFqeFuaaa@3841@ contains two pins (*q*_1 _and *q*_*Z*_), S
 MathType@MTEF@5@5@+=feaafiart1ev1aaatCvAUfKttLearuWrP9MDH5MBPbIqV92AaeXatLxBI9gBamrtHrhAL1wy0L2yHvtyaeHbnfgDOvwBHrxAJfwnaebbnrfifHhDYfgasaacH8akY=wiFfYdH8Gipec8Eeeu0xXdbba9frFj0=OqFfea0dXdd9vqai=hGuQ8kuc9pgc9s8qqaq=dirpe0xb9q8qiLsFr0=vr0=vr0dc8meaabaqaciaacaGaaeqabaWaaeGaeaaakeaaimaacqWFse=uaaa@3845@(*i*) is the full *N *nucleotides of **y**, and the algorithm computes the full unconstrained structural alignment algorithm.

Each constraint provided is an additional pin in Q
 MathType@MTEF@5@5@+=feaafiart1ev1aaatCvAUfKttLearuWrP9MDH5MBPbIqV92AaeXatLxBI9gBamrtHrhAL1wy0L2yHvtyaeHbnfgDOvwBHrxAJfwnaebbnrfifHhDYfgasaacH8akY=wiFfYdH8Gipec8Eeeu0xXdbba9frFj0=OqFfea0dXdd9vqai=hGuQ8kuc9pgc9s8qqaq=dirpe0xb9q8qiLsFr0=vr0=vr0dc8meaabaqaciaacaGaaeqabaWaaeGaeaaakeaaimaacqWFqeFuaaa@3841@ and reduces the range of indices which must be considered. Figure 3 panel B outlines the constrained structural alignment algorithm in pseudocode. The existence of alignment pins does not restrict the structure prediction (indices *i*, *a*, and *j*) of the first sequence, **x**. However, for a particular instance of each of these indices, the corresponding range of analogous positions in **y **(*k*, *b*, and *l *respectively) is reduced. If we were using an ungapped alignment approach, a single midpoint pin would reduce the range of indices in **y **by 1/2, effectively reducing the memory by 1/4 and the runtime by 1/8.

When a position of interest *i *is a pin, the precise alignment partner is known. It might seem that the segment S
 MathType@MTEF@5@5@+=feaafiart1ev1aaatCvAUfKttLearuWrP9MDH5MBPbIqV92AaeXatLxBI9gBamrtHrhAL1wy0L2yHvtyaeHbnfgDOvwBHrxAJfwnaebbnrfifHhDYfgasaacH8akY=wiFfYdH8Gipec8Eeeu0xXdbba9frFj0=OqFfea0dXdd9vqai=hGuQ8kuc9pgc9s8qqaq=dirpe0xb9q8qiLsFr0=vr0=vr0dc8meaabaqaciaacaGaaeqabaWaaeGaeaaakeaaimaacqWFse=uaaa@3845@(*i*)should therefore be one nucleotide, but this is only true in ungapped alignments. In gapped alignments, we must consider that the pin may end or begin a gap. An indel aligns a nucleotide to *nothing *and in the dynamic programming algorithm one index *k *progresses while the other *i *is fixed. Consequently, to consider all possible gap states requires S
 MathType@MTEF@5@5@+=feaafiart1ev1aaatCvAUfKttLearuWrP9MDH5MBPbIqV92AaeXatLxBI9gBamrtHrhAL1wy0L2yHvtyaeHbnfgDOvwBHrxAJfwnaebbnrfifHhDYfgasaacH8akY=wiFfYdH8Gipec8Eeeu0xXdbba9frFj0=OqFfea0dXdd9vqai=hGuQ8kuc9pgc9s8qqaq=dirpe0xb9q8qiLsFr0=vr0=vr0dc8meaabaqaciaacaGaaeqabaWaaeGaeaaakeaaimaacqWFse=uaaa@3845@(*i *= *q*_*z *_(*x*)) to imply *q*_*z*-1 _(*y*) <*k *<*q*_*z*+1 _(*y*). In this case the segment S
 MathType@MTEF@5@5@+=feaafiart1ev1aaatCvAUfKttLearuWrP9MDH5MBPbIqV92AaeXatLxBI9gBamrtHrhAL1wy0L2yHvtyaeHbnfgDOvwBHrxAJfwnaebbnrfifHhDYfgasaacH8akY=wiFfYdH8Gipec8Eeeu0xXdbba9frFj0=OqFfea0dXdd9vqai=hGuQ8kuc9pgc9s8qqaq=dirpe0xb9q8qiLsFr0=vr0=vr0dc8meaabaqaciaacaGaaeqabaWaaeGaeaaakeaaimaacqWFse=uaaa@3845@(*i*) overlaps the segments S
 MathType@MTEF@5@5@+=feaafiart1ev1aaatCvAUfKttLearuWrP9MDH5MBPbIqV92AaeXatLxBI9gBamrtHrhAL1wy0L2yHvtyaeHbnfgDOvwBHrxAJfwnaebbnrfifHhDYfgasaacH8akY=wiFfYdH8Gipec8Eeeu0xXdbba9frFj0=OqFfea0dXdd9vqai=hGuQ8kuc9pgc9s8qqaq=dirpe0xb9q8qiLsFr0=vr0=vr0dc8meaabaqaciaacaGaaeqabaWaaeGaeaaakeaaimaacqWFse=uaaa@3845@(*i *- 1) and S
 MathType@MTEF@5@5@+=feaafiart1ev1aaatCvAUfKttLearuWrP9MDH5MBPbIqV92AaeXatLxBI9gBamrtHrhAL1wy0L2yHvtyaeHbnfgDOvwBHrxAJfwnaebbnrfifHhDYfgasaacH8akY=wiFfYdH8Gipec8Eeeu0xXdbba9frFj0=OqFfea0dXdd9vqai=hGuQ8kuc9pgc9s8qqaq=dirpe0xb9q8qiLsFr0=vr0=vr0dc8meaabaqaciaacaGaaeqabaWaaeGaeaaakeaaimaacqWFse=uaaa@3845@(*i *+ 1), shown in Figure 3 panel C. For this reason, a single midpoint pin takes more memory and longer time than the ungapped case.

### Testing

Our work on single sequence grammars indicated that prediction accuracy can vary significantly between RNA families [37]. The recent benchmark of Gardner et. al. utilizes only a set of tRNA alignments to compare pairwise structural alignment algorithms [45]. We desired to extend this test set to multiple RNA families, but are limited to small RNAs to facilitate comparison to the unconstrained Sankoff algorithm. Therefore we use as our primary test data 1114 tRNA and 602 5S RNA sequences in the Rfam v7.0 seed alignments [46]. Different subsets of these data were generated to examine particular aspects of the pairSCFG, as described in Results.

We also obtained published test sets from David Mathews[30,35] and Ian Holmes[31] to facilitate direct comparison to Dynalign and Stemloc. The Mathews sets consist of tRNA, SRP and 5S RNA sequences, where each sequence has a known secondary structure but alignment information is not given. The Holmes dataset consists of sequences from Rfam v6.1 seed alignments spanning seven different families. The pairs utilized have published consensus secondary structure and no pair is higher than 60% identical. The set includes 2 S15 pairs, 1 U3 pair, 5 glmS riboswitch pairs, 4 Purine riboswitch pairs, 2 U5 pairs, 6 IRE pairs, and 2 6S RNA pairs, for a total of 22 pairwise comparisons.

We utilize base pair sensitivity and base pair positive predictive value (PPV) as structure prediction accuracy measures [38]. We compare the structure predicted by the pairSCFG to the structure given in the trusted alignment. We report sensitivity and PPV as cumulative statistics over all possible base pairs (total correct base pairs/total base pairs in all pairs) within the testset.

We calculate the alignment identity between the trusted pairwise structural alignment and our predicted alignment, defined as the number of alignment columns correctly determined relative to the trusted (given) alignment. As with structure comparisons, we compute alignment identity cumulatively over all columns in all alignment pairs.

We determine the standard deviation of all three measures by bootstrapping using 10,000 samples[47].

### Implementation

Parameter estimation (training) and the CYK algorithm was written for both the pairSCFG and its corresponding single sequence grammar *G*_*s*_. The constrained CYK algorithm was implemented for the pairSCFG in ANSI C. The source code for the package, called Consan, is freely available under the GNU General Public License (GPL) from [48]. The training and test data are freely available from the same URL.

To determine pins, we utilize Ian Holmes' *dpswalign *program, a pairHMM implementation from his Dart package v0.2[49]. The program is used with default parameters. Two options are utilized, the "-pt" option returns the posterior probability table between any two sequences and the "-oa" option returns the optimal accuracy alignment.

For benchmarking experiments, we use *mfold *v3.1.2, clustalw v1.83, Dynalign (Sept 2004), and *Stemloc *from the Dart software package v0.2 (Oct 2004), RNAfold from the ViennaRNA v1.6 package, the PMcomp.pl script (Nov 2004), and FOLDALIGN v2.0.3. Dynalign is used with the parameters suggested in [30], Stemloc uses the parameters described in [31], PMcomp and FOLDALIGN are utilized as described in [45]. Benchmarks were conducted on a a dual 2.8 GHz P4 Linux machine with 2.5 GB of memory.

### Pairwise structure prediction improves relative to single sequence

In earlier work [37], we showed that small single sequence grammars similar to G_*s *_have only slightly worse performance than standard minimum energy methods. In order to verify that the new G_*s *_single-sequence grammar design performs similarly to the previously tested small grammars, we trained it using the rRNA training set and compare its performance to *mfold *for single sequence folding on a test set of 100 tRNA and 100 5S sequence randomly chosen from the Rfam v7.0 seed alignments. As shown in Table 1, the G_*s *_grammar shows the expected performance lag relative to *mfold*, a necessary trade-off for a simpler grammar[37].

**Table 1:** Comparing structure prediction performance of single sequence methods to pairSCFG. A comparison of the single sequence G_*s *_grammar and *mfold *to the pairSCFG.

	Full Set		5S		tRNA	
Method	Sens	PPV	Sens	PPV	Sens	PPV
mfold v3.1.2	58 ± 2	56 ± 2	52 ± 3	49 ± 3	65 ± 3	62 ± 3
G_*s*_	48 ± 2	51 ± 2	39 ± 2	42 ± 2	61 ± 3	65 ± 3
self vs self G_*s*_	47 ± 2	42 ± 2	38 ± 3	35 ± 2	61 ± 3	55 ± 3
pairwise	67 ± 2	68 ± 2	56 ± 2	57 ± 2	85 ± 2	84 ± 2

Using the pairSCFG to compare a sequence to itself (self vs self) shows that the pairwise grammar performs roughly equivalent to its single sequence counterpart in the absence of alignment information.

As a control experiment to verify that the extension to a pairSCFG does not significantly alter single sequence prediction accuracy (and that we had not made any glaring implementation errors), we evaluated the pairSCFG on "pairwise alignments" of the test sequences to themselves, with the expectation that it should show similar performance to the single sequence grammar G_*s*_.

We then tested whether the corresponding pairSCFG improved structure prediction accuracy on the same test set, using the unconstrained pairSCFG CYK algorithm. We trained the pairSCFG on all pairs of the rRNA training set. Using the same 5S and tRNA test set, we randomly selected an "informant" sequence from the 5S and tRNA seed alignments within the 55–80% identity range emphasized by the rRNA training set. In evaluation, however, we only consider the ability to predict the original test sequence's structure.

Table 1 shows that, using a homologous informant sequence for pairwise comparison, the pairSCFG significantly improves the structure prediction accuracy relative to single sequence prediction by either *mfold *or G_*s*_. On self vs. self identical alignments, the pairSCFG gives similar basepair sensitivity as the single sequence grammar G_*s *_on which it was based, as expected. PPV performance drops, because the pairSCFG overcalls base pairs in these identical alignments. Its parameterization has learned that base pairs are often more conserved than single stranded residues in homologous alignments.

### Structure prediction accuracy depends on pairwise sequence identity

We expect that the performance of any pairwise structural alignment algorithm will depend on the similarity of the sequence pair. Closely related pairs will lack sufficient covariation to differ from single sequence structure prediction. Pairs at too great a distance may be difficult to align even when secondary structure information is taken into account.

To test the effects of sequence identity on both structure and alignment performance, we built a series of test sets binned by pairwise identity. The Rfam v7.0 seed alignment sequence pairs for 5S and tRNA are divided into 20 bins, each representing a 5% identity interval for the known structural alignment. Within each bin 10 sequence pairs are selected at random for both 5S and tRNA. Because sequence pairs were unavailable at the lowest percent identities, the resulting test set contains 184 tRNA pairs between 9% and 99% identical and 140 are 5S pairs between 32% and 99% identical.

**Figure 4:**
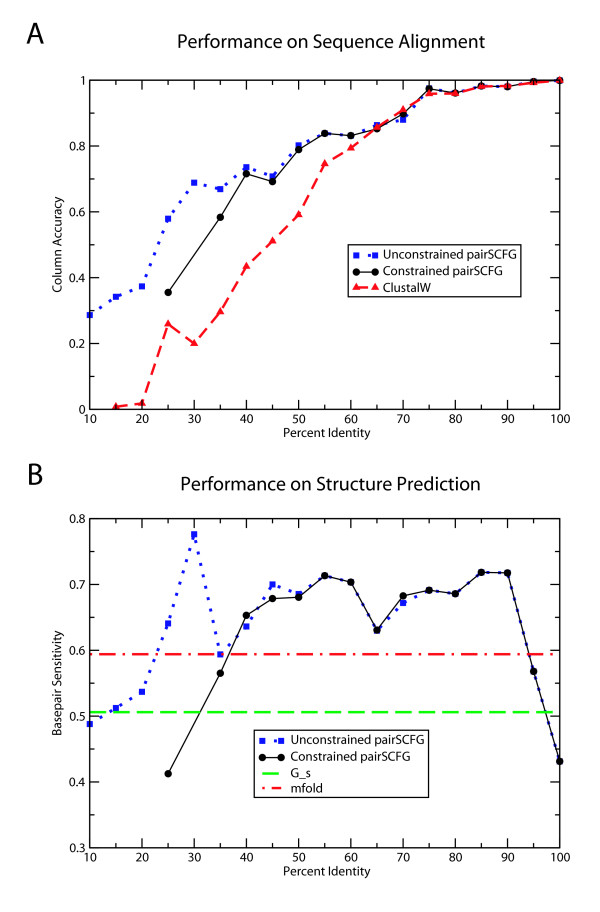
**Performance of pairSCFG algorithm on test sets binned by percent identity**. *Panel A *shows the alignment accuracy for the unconstrained pairSCFG (blue squares; dotted line), the constrained pairSCFG (black circles; solid line), and clustalW (red triangles; dashed line). *Panel B *shows the base pair sensitivity of the unconstrained pairSCFG (blue squares; dotted line) and constrained pairSCFG (black circles; solid line). For reference, the performance of both G_*s *_(dashed green line) and *mfold *(dashed red line) on this set of sequences is shown. In both graphs, the performance of the constrained pairSCFG is only given for percent identity bins where at least two alignments contained valid pins. All comparisons are relative to the Rfam v7.0 seed alignments.

We then utilize these binned sets to compare the pairwise alignment accuracy of the pairSCFG to ClustalW, shown in Figure 4A. Alignment accuracy improves as sequence identity increases, as expected, and the pairSCFG is a more robust aligner at lower sequence identities.

We also examined the structure prediction accuracy of the pairSCFG over a range of sequence identities. For comparison to *mfold *and the single sequence grammar G_*s*_, we extract all individual sequences and determined the base pair sensitivity on this collective set. These results are shown in Figure 4B.

For structure prediction, the accuracy of the pairSCFG at low (<25%) and high (>90% identity) is not strikingly different than single sequence prediction by *mfold *or G_*s*_. For sequence pairs between 40 and 90% identity, the pairSCFG improves structure prediction with respect to *mfold *by about 10% on average for 5S and about 20% for tRNA.

### The unconstrained algorithm is compute-intensive

Although the alignment and structure prediction results above are promising, the runtime and memory requirements of the unconstrained pairSCFG algorithm are extreme. For two representative tRNAs, lengths 76 and 77, the algorithm requires 290 MB of memory and 910 seconds. For two representative 5S sequences, lengths 116 and 117, the algorithm requires 1556 MB of memory and 22,902 seconds.

### Generating pins for the constrained algorithm

Figure 4 shows that the pairSCFG performs the best relative to single sequence RNA structure prediction for moderately diverged sequence pairs sharing between 40 and 90% identity. For moderately diverged sequences like this, primary sequence alignment alone will usually be able to identify some regions of confident alignment. We therefore adopt a two-stage heuristic strategy in which we first use a probabilistic (pairHMM) primary sequence alignment to identify *pins *(confidently aligned residue pairs), and then use those pins in a constrained Sankoff pairSCFG CYK algorithm.

We think we can afford to focus on comparisons within a particular broad range of similarity because comparative genome sequencing has advanced so that there are often many sequence homologs available at different levels of similarity. We usually have some freedom to pick and choose "optimal" homologs for assisting an RNA secondary structure prediction.

**Figure 5:**
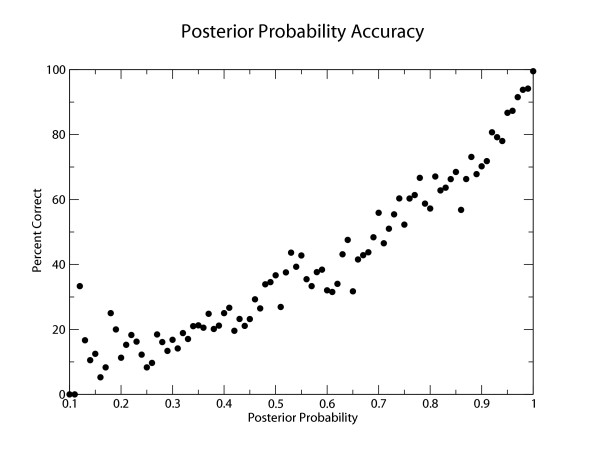
**Accuracy of Posterior Probabilities**. A graph of the posterior probabilities of optimal accuracy alignment positions (X axis) against the percentage of these pins which are correct relative to the known structural alignment.

To obtain a set of pins consistent with a single alignment, and to rank them by reliability, we use a pairHMM to calculate a posterior probability for each aligned pair of residues in a pairwise sequence alignment, using Ian Holmes' *dpswalign *program in his Dart package, and we calculate an "optimal accuracy" alignment that maximizes the sum of these posteriors [49]. In principle, we expect that a 90% posterior probability pair should be correct 90% of the time. To test how well posterior probabilities are actually correlated with correct alignment, using the same test sets as above binned by percent identity, we collected the calculated posterior probability for all aligned residue pairs in all pairwise alignments in each bin, and assessed their correctness relative to the known structural alignment. The result is shown in Figure 5. In optimal accuracy alignments, the calculated posterior probabilities predict empirical alignment accuracy reasonably well. 99% of pairs with 1.0 posteriors are correct, and 93% of pairs with posteriors between .95 and 1.0 are correct.

We then select a subset of "quality" pins from the set of aligned residues in the optimal accuracy alignment. Pin selection is a trade-off. With each additional pin, the constrained algorithm will take less memory and time; however, because pins are fixed, an incorrect pin choice cannot be rectified later by the constrained algorithm. Additionally, pins that are evenly spaced provide more of a performance gain, as opposed to pins that are tightly clustered. One method of enforcing pin spacing is to define a window around each pin in which no other pins can be selected.

We examined a number of different combinations of posterior probability cutoffs between 0.80 and 1.0 and "protection windows" between 0 and 30 nucleotides using a pin selection strategy which is greedy, always selecting the best available pin given the protection window. Regardless of the posterior threshold, with short protection windows (≤ 15 nucleotides) incorrect pins are detected in many pairs, including those with high percent identity (i.e. a 93% identical pair). The average compute resource requirements (runtime and memory) increase with window length, as this is the primary determinant of the number of pins possible. For example, at a protection window of 20 nucleotides, the average number of pins found for the set of 5S pairs containing at least one pin is 4.3 and the average runtime is approximately 219 seconds. Increasing the window to 25 nucleotides decreases the average pins found to 3.6 which results in a average runtime of 259 seconds. At a particular window length, the posterior probability threshold affects both the number of sequence pairs for which any pin can be found and the ability of the constrained algorithm to reproduce the unconstrained results.

The settings for the posterior threshold and window length parameters trade off compute performance against alignment and structure prediction accuracy. Table 2 shows, as an example, results of changing the posterior probability threshold on the tRNA subset of the testset using a fixed window length of 20 nucleotides. Results for the 5S subset and other window lengths have similar trends (not shown). Reducing the posterior threshold increases the number of sequence pairs containing at least one pin, and thus increases the number and dissimilarity of sequence pairs that can be aligned in constrained mode. In this experiment, the performance of the constrained algorithm is not statistically different from the unconstrained algorithm for any of the examined posterior cutoffs; however, Figure 5 indicates that a posterior of 0.90 is only correct about 75% of the time. Therefore 1 of every 4 pins selected at a 0.90 threshold would be expected to be incorrect, so it would not be wise to reduce the threshold too far, for fear of introducing incorrect pins. We chose a default pin selection strategy of greedily selecting pins greater than 0.95 posterior probability with a 20 nucleotide protection window. A user might need to reduce the default posterior threshold to align highly dissimilar sequences; we see little reason for a user to alter the protection window parameter.

**Table 2:** Varying Pin Selection Posterior Cutoff. Effects of varying the posterior probability cutoff at a fixed window length of 20 nucleotides, for all pairs of tRNA sequences in the test set.

Selection Criteria	Num Pairs	Lowest % ID	Full Sankoff		Constrained		Time (s)		Memory (MB)	
			BP Sens	Align ID	BP Sens	Align ID	Avg	Longest	Avg	Longest
tRNA subset										

no pins	184	9	77 ± 2	83 ± 2	-	-	570	2674	255	560
= 1.0	12	56	88 ± 3	92 ± 3	88 ± 3	92 ± 3	97	197	137	226
≥ 0.99	115	35	84 ± 2	94 ± 1	84 ± 2	94 ± 1	34	218	69	236
≥ 0.98	124	18	84 ± 2	93 ± 1	84 ± 2	93 ± 1	32	240	72	236
≥ 0.95	135	14	83 ± 2	91 ± 1	82 ± 2	91 ± 1	32	171	70	232
≥ 0.90	146	10	81 ± 2	88 ± 2	80 ± 2	87 ± 2	33	145	72	232
≥ 0.85	152	10	80 ± 2	87 ± 2	79 ± 2	86 ± 2	31	241	69	232
≥ 0.80	158	9	80 ± 2	86 ± 2	79 ± 2	85 ± 2	31	139	69	214

For higher posterior thresholds, there are fewer sequence pairs in which at least one pin can be identified; the number of sequence pairs that can be pinned and aligned is indicated in the Num Pairs column, and the percent identity of the most dissimilar pair that can be pinned is indicated in the Lowest ID column. For the subset of pairs that can be pinned at a given posterior threshold, we evaluate basepair sensitivity (BP Sens) and alignment identity (Align ID) for full Sankoff (unconstrained) alignment and the Constrained (pinned) alignment. Comparing these numbers within each row indicates the performance impact of the constraints. The average and maximum resource requirements (Time and Memory) are also shown for each posterior cutoff. The performance gain from pins is primarily determined by the number of pins assigned in a sequence pair. For the subset of sequence pairs that can be pinned at all, the average number of pins is largely a function of the window length and somewhat independent of posterior threshold (here W = 20; avg pins = 2.6). The maximum time and memory requirements are typically for a sequence pair in which only a single pin was discovered.

### Pin number and compute requirements depend on pairwise sequence identity

**Figure 6:**
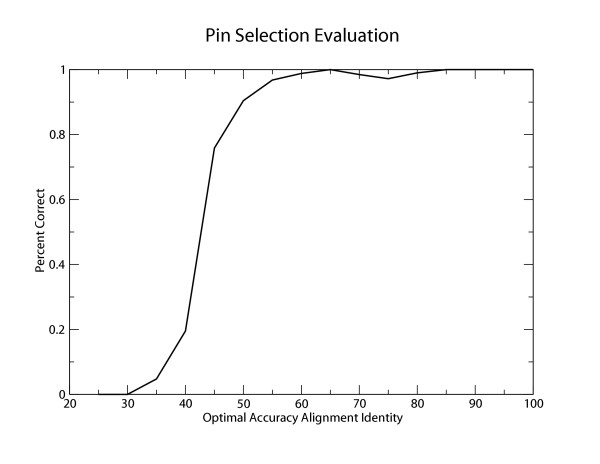
**Pin Selection Evaluation**. Using the optimal accuracy alignment and a fixed protection window of 20, we determine the correctness of selected pins given our posterior cutoff (> 0.95).

Having decided on this strategy, we next evaluated how many pins are generated for sequences of different levels of identity. The availability of at least one quality pin correlates roughly with the percent identity of the sequence pair. All pairs > 45% identical have at least 2 quality pins whereas only 4 pairs < 30% identical have 1 quality pin apiece. Furthermore, 82/324 pairs find the maximum number of pins (6 for 5S and 4 for tRNA) permissible given the length of the sequences and a protection window of 20 nucleotides. We then asked how many of these selected pins are correct relative to the structural alignment (Figure 6). These results show that above about 50% pairwise identity, we can identify accurate pins.

Finally, we compare the constrained pairSCFG to the unconstrained pairSCFG using the previously described percent identity binned test set. The results are shown in Figure 4 for both alignment identity and base pair sensitivity, where points are only included on the graph if at least two pairs within the bin have pins meeting our quality criteria. The availability of pins divides roughly into three regions based on percent identity. For low percent identity pairs (< 35%) only 5/44 pairs have quality pins. At slightly higher percent identities, between 35 and 45% identical, pins are found for roughly half of the pairs and the performance of the constrained algorithm is slightly worse than the unconstrained algorithm. For pairs greater than 45% identical, pins are found for all pairs and constrained pairSCFG has performance nearly identical to the unconstrained algorithm.

In general, the runtime and memory requirements of the constrained algorithm are dominated by the largest unpinned segment. Performance depends on the distribution of the pins and optimal performance is achieved with evenly spaced pins. Even a single pin, though, reduces the search space. For two 90 mers, the unconstrained algorithm requires 560 MB memory and 3438 seconds to compute a structural alignment. A single central pin reduces this to 210 MB and 255 seconds.

### Comparison to other methods

We know of four constrained Sankoff implementations, Dynalign [30,35], Stemloc [28,31], PMcomp[36], and FOLDALIGN [34,32] which attempt to solve the same pairwise alignment and structure prediction problem as Consan does.

#### Dynalign

Dynalign is essentially a pairwise extension to the thermodynamic single sequence program RNAstructure. Dynalign computes an alignment and consensus secondary structure that minimizes the sum of the predicted folding energies of the two individual sequences. It adds an ad hoc pseudoenergy penalty for insertions and deletions, but otherwise does not score the sequence alignment. It constrains the alignment by assuming that it is a global alignment of sequences of similar length, and restricts all aligned residue pairs to have indices differing by no more than a given maximum.

In his most recent paper describing Dynalign [35], Mathews utilizes four datasets to evaluate its performance. The first contains 7 5S sequences selected for comparison to the earlier Dynalign implementation [30]. The second contains three pairs of SRP sequences to assess performance on longer sequences. The last two are randomly selected sets to assess performance without selection bias, containing 40 tRNA and 14 5S sequences. As provided by Mathews, these datasets contain only individual sequences and their secondary structures, not pairwise or multiple alignments, so alignment accuracy could not be assessed on these test sets. When evaluating Dynalign, we examine each pair once, excluding self to self comparisons. Because the two 5S datasets gave comparable results, we combined their results and report a single 5S metric. Using our benchmark procedure Dynalign obtains basepair sensitivity measures of 82 ± 5 on 5S, 81 ± 2 on SRP, and 86 ± 2 on tRNA.

We utilize the constrained algorithm with the default pin selection criteria (posterior > 0.95, protection window = 20). For 8/112 5S pairs and 106/781 tRNA pairs, no pins met our selection criteria and Consan falls back to an unconstrained Sankoff algorithm. With these criteria, our algorithm obtains sensitivity measures of 74 ± 2 on 5S, 79 ± 5 on SRP, and 87 ± 2 on tRNA. Thus, Dynalign performs slightly better on 5S RNAs, and on the other two sets the methods perform about the same.

#### Stemloc

Ian Holmes' *Stemloc *is comparable in several respects to our work. Stemloc is also based on a pairSCFG, though its grammar is quite different from the grammar in Consan. Holmes' constrained algorithm is based on the general concept of fold and alignment envelopes [28,31]. Our pins are essentially a special case of an alignment envelope. Stemloc computes a fold envelope from the union of many possible individual foldings of each sequence according to a single sequence SCFG, and it computes an alignment envelope from the union of many possible alignments of the two sequences according to a pairHMM. The pairSCFG then only considers solutions which are consistent with these precomputed fold and alignment envelopes [31].

Holmes' test dataset consists of 22 pairs of sequences from Rfam seed alignments. He reports a single basepair sensitivity and alignment accuracy value for each pair, using default parameters of 100 alignments and 1000 folds. Because Stemloc is not scoring symmetric, we examine all pairs, excluding self-to-self. Using our benchmark procedure, Stemloc obtains 65 ± 4 sensitivity, 61 ± 4 PPV and 71 ± 3 alignment accuracy. Using our constrained pairSCFG, (comparing each pair only once) we achieve 67 ± 4 sensitivity, 61 ± 5 PPV and 71 ± 4 alignment accuracy on the same dataset. Thus the two pairSCFG implementations show comparable performance on Stemloc's test dataset.

#### Comparisons on our test dataset

We compared the five constrained Sankoff implementations on our test set binned by percent identity. We compare each pair only once, with the sequence order being determined randomly. For Dynalign, we used the parameters described in [35], excluding two pairs which caused the program to crash. With Stemloc, we used the parameters suggested by [31], except when insufficient memory was available. In these cases we stepped down the number of folds (-nf) gradually (by 100s) until we could run the pair. PMcomp is utilized without the fast option, as described by its documentation and as was utilized by Gardner[45]. One pair was excluded, as PMcomp's backtrack fails for the pair. FOLDALIGN is utilized as described by Gardner[45] (-global -max_diff 25 -score_matrix global.fmat). For Consan, we use the default constrained method when pins are available and the unconstrained algorithm when no pins meet our quality criteria (> 0.95 posterior and protection window = 20).

**Figure 7:**
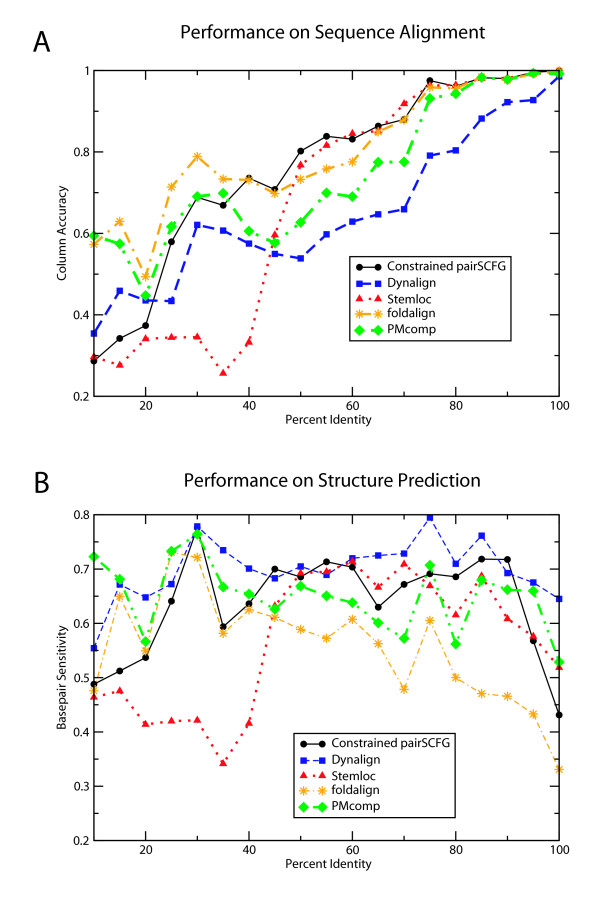
**Performance of constrained Sankoff implementations on test sets binned by percent identity**. Each bin represents 10% identity and contains 10 randomly selected pairwise comparisons. The top panel (A) shows the alignment accuracy of Consan (circles, black line), Dynalign (squares, blue line), Stemloc (triangles, red line), FOLDALIGN (stars, orange line), and PMcomp (diamonds, green line). The lower panel (B) shows the structure prediction accuarcy of the same implementations.

Figure 7 shows the performance of the five implementations on alignment prediction (A) and basepair sensitivity (B) at each percent identity. In general, the five methods are comparable, with a few features worth noting. Stemloc, FOLDALIGN, and Consan produce better alignments over most identities, presumably because they score a combination of alignment and consensus structure, whereas Dynalign and PMcomp almost exclusively scores the consensus structure alone. Dynalign is generally best at structure prediction over the widest range. At the highest and lowest identities, where we expect sequence alignment to be either uninformative or impossible, the strength of the well-developed thermodynamic model for RNA folding shines, as demonstrated by Dynalign and PMcomp. The general trends demonstrated by this benchmark are consistent with those reported by Gardner[45].

Despite both using pairSCFG approaches, Consan appears to produce better alignments and better structures over a wider identity range than Stemloc. We can think of at least two hypotheses for this effect. First, because Stemloc currently calculates alignment envelopes defined by complete pairwise alignments, its performance might suffer at low percent identities where obtaining any accurate complete primary sequence alignment is difficult, even in a large sample of suboptimal alignments. We may be more robust to this effect by constraining only on a subset of confident pins. Second, although the Stemloc grammar was said to be both structurally unambiguous and alignment unambiguous, we think it is in fact alignment ambiguous, meaning that multiple parse trees can correspond to the same set of aligned residue pairs. This might have a negative impact on accuracy at low identities, because an ambiguous grammar does not rank suboptimal alignments correctly by their probabilities.

In terms of computational resources, the Dynalign algorithm utilizes a banding approach that reduces the algorithm to *O*(*N*^3^ℳ
 MathType@MTEF@5@5@+=feaafiart1ev1aaatCvAUfKttLearuWrP9MDH5MBPbIqV92AaeXatLxBI9gBamrtHrhAL1wy0L2yHvtyaeHbnfgDOvwBHrxAJfwnaebbnrfifHhDYfgasaacH8akY=wiFfYdH8Gipec8Eeeu0xXdbba9frFj0=OqFfea0dXdd9vqai=hGuQ8kuc9pgc9s8qqaq=dirpe0xb9q8qiLsFr0=vr0=vr0dc8meaabaqaciaacaGaaeqabaWaaeGaeaaakeaaimaacqWFZestaaa@3790@^3^) in time and *O*(*N*^2^ℳ
 MathType@MTEF@5@5@+=feaafiart1ev1aaatCvAUfKttLearuWrP9MDH5MBPbIqV92AaeXatLxBI9gBamrtHrhAL1wy0L2yHvtyaeHbnfgDOvwBHrxAJfwnaebbnrfifHhDYfgasaacH8akY=wiFfYdH8Gipec8Eeeu0xXdbba9frFj0=OqFfea0dXdd9vqai=hGuQ8kuc9pgc9s8qqaq=dirpe0xb9q8qiLsFr0=vr0=vr0dc8meaabaqaciaacaGaaeqabaWaaeGaeaaakeaaimaacqWFZestaaa@3790@^2^) in memory where ℳ
 MathType@MTEF@5@5@+=feaafiart1ev1aaatCvAUfKttLearuWrP9MDH5MBPbIqV92AaeXatLxBI9gBamrtHrhAL1wy0L2yHvtyaeHbnfgDOvwBHrxAJfwnaebbnrfifHhDYfgasaacH8akY=wiFfYdH8Gipec8Eeeu0xXdbba9frFj0=OqFfea0dXdd9vqai=hGuQ8kuc9pgc9s8qqaq=dirpe0xb9q8qiLsFr0=vr0=vr0dc8meaabaqaciaacaGaaeqabaWaaeGaeaaakeaaimaacqWFZestaaa@3790@ is the maximum distance[30,35] between any two positions in the alignment. Stemloc uses precomputed alignment envelopes and/or fold envelopes to reduce the search space of the structural alignment. The pre-computation steps are *O*(*N*^2^) for alignment envelopes and *O*(*N*^3^) for fold envelopes in time, and *O*(*N*^2^) in memory for both. The final structural alignment phase remains *O*(*N*^6^) and *O*(*N*^4^) in time and space respectively. PMcomp uses precomputed pair probability matrices, computed in *O*(*N*^3^) in time. The structural alignment phase uses a structural banding approach, restricting the difference in the span of matching base pairs to Δ which reduces the computational effort to *O*(*N*^3^) in memory and *O*(*N*^4^) in time. FOLDALIGN uses both alignment banding (*δ*) and structural banding (*λ*) to reduce the time complexity to *O*(*N*^2^*λ*^2^*δ*^2^) and the memory complexity to *O*(*λ*^3^*δ*). Our pinned approach calculates its pins in *O*(*N*^2^) time and space and the structural alignment remains in general *O*(*N*^6^)and *O*(*N*^4^) in time and space; in the limit of an alignment where all positions are pins, our constrained algorithm reduces to *O*(*N*^3^) in time and *O*(*N*^2^) in space.

Table 3 shows examples of the empirical resource requirements of six Sankoff implementations: our unconstrained structural alignment algorithm, our constrained alignment algorithm, Stemloc, Dynalign, PMcomp, and FOLDALIGN. Dynalign is about an order of magnitude slower than the other implementations, but somewhat less memory hungry. (It should be noted that, while this paper was in review, an update to the Dynalign algorithm was reported which improves its runtime performance[33].) Of the two pairSCFG algorithms, Stemloc is significantly faster and uses about the same memory as Consan.

**Table 3:** Resource Utilization Comparison.

Sequence Pair	CPU (sec)	RAM (MB)
unconstrained pairSCFG		

RD0260 vs RE6781 tRNAs	718	285
M16173 vs X02128 5S	22902	1465

constrained pairSCFG (post 0.95; W = 20)		

RD0260 vs RE6781 tRNAs	31	64
M16173 vs X02128 5S	347	248

Dynalign (ℳ MathType@MTEF@5@5@+=feaafiart1ev1aaatCvAUfKttLearuWrP9MDH5MBPbIqV92AaeXatLxBI9gBamrtHrhAL1wy0L2yHvtyaeHbnfgDOvwBHrxAJfwnaebbnrfifHhDYfgasaacH8akY=wiFfYdH8Gipec8Eeeu0xXdbba9frFj0=OqFfea0dXdd9vqai=hGuQ8kuc9pgc9s8qqaq=dirpe0xb9q8qiLsFr0=vr0=vr0dc8meaabaqaciaacaGaaeqabaWaaeGaeaaakeaaimaacqWFZestaaa@3790@ = 15; gap = 0.4)		

RD0260 vs RE6781 tRNAs	1761	33
M16173 vs X02128 5S	4928	67

Stemloc (-na 100 -nf 1000)		

RD0260 vs RE6781 tRNAs	6	85
M16173 vs X02128 5S	18	193

PMcomp (-p -noLP for RNAfold)		

RD0260 vs RE6781 tRNAs	19	52
M16173 vs X02128 5S	33	142

FOLDALIGN (-max_diff 25 -global -score_matrix global. fmat)		

RD0260 vs RE6781 tRNAs	25	77
M16173 vs X02128 5S	87	280

The resource requirements for a single representative tRNA and 5S sequence pair for each of the five constrained Sankoff implementations and the unconstrained pairSCFG Sankoff implementation, given as a baseline reference.

The tRNA sequences are of length 78 and 77 respectively. The 5S sequences are of length 117 and 118 respectively. The constrained pairSCFG utilizes the pin selection criteria of posteriors > 0.95 and a protection window of 20. (For these examples the result is 2 pins for the tRNA pair and 3 pins for the 5S pair.) Dynalign is compared using the parameters recommended in [35] of ℳ
 MathType@MTEF@5@5@+=feaafiart1ev1aaatCvAUfKttLearuWrP9MDH5MBPbIqV92AaeXatLxBI9gBamrtHrhAL1wy0L2yHvtyaeHbnfgDOvwBHrxAJfwnaebbnrfifHhDYfgasaacH8akY=wiFfYdH8Gipec8Eeeu0xXdbba9frFj0=OqFfea0dXdd9vqai=hGuQ8kuc9pgc9s8qqaq=dirpe0xb9q8qiLsFr0=vr0=vr0dc8meaabaqaciaacaGaaeqabaWaaeGaeaaakeaaimaacqWFZestaaa@3790@ = 15 and a gap penalty of 0.4 kcal mol^-1 ^gap^-1^. Stemloc is compared using the parameters recommended in [31] which computes 100 alignment envelopes (-na 100) and 1000 fold envelopes (-nf 1000). For PMcomp, the reported time includes both calculating the pair probability (via RNAfold with parameters -p -noLP) and the subsequent pmcomp.pl phase. The memory reported is the maximum utilized during either phase. With FOLDALIGN, the parameters from Gardner are utilized (-max_diff 25-global -score_matrix global.fmat). For all programs these are the same parameters utilized to generate the performance reflected in Figure [7]. These benchmarks were conducted on a dual 2.8 GHz P4 machine with 2.5 GB memory running the Linux 2.4 kernel.

## Conclusion

Stochastic context-free grammars provide a unifying framework for simultaneously scoring of alignment and secondary structure folding, providing a strong formal basis for scoring systems in comparative RNA secondary structure applications [39]. Holmes' Stemloc and our Consan both use pairSCFGs as the basis for an implementation of the Sankoff algorithm for pairwise RNA structural alignment. The two implementations differ primarily in two respects. First, they use different methods of heuristically constraining the dynamic programming algorithm to make it practical. Second, they use different underlying grammar designs.

Stemloc's concept of alignment and fold envelopes is a general one, and the concept includes our pins as a special case. As Holmes notes [31], there are many ways one could imagine determining the allowed envelope. As implemented, Stemloc relies on the union of a finite sample of suboptimal folds and alignments to define its envelopes. Our simpler alignment pinning strategy is less general, but it may have certain advantages when the complete alignment cannot be identified reliably even in this union of suboptimals, but parts of it can be reliably pinned.

Grammar design issues are of great interest to us. Earlier work [37] showed that grammar design can have significant impact on the performance on secondary structure prediction. We developed our pairSCFG by extending a small but good single sequence SCFG design. We believe that it is important for SCFG designs to be structurally and alignment-unambiguous, as we described in the Methods. We do not believe the Stemloc design is alignment unambiguous, and we think (but have not proven) that Consan's somewhat better performance at lower percent identities might be due in part to grammar ambiguity issues.

Be that as it may, we do not think that either the Stemloc or the Consan grammar design will prove to be the best for this problem. Both implementations use relatively simple grammars that do not approach the descriptive power of the current thermodynamic rules for RNA folding. For example, neither grammar has a model of explicit loop lengths akin to the hairpin, bulge, and interior loop length tables of RNA folding thermodynamic rules; nor do they model nearest-neighbor base pair stacking correlations as the energy tables do. Between our work and Holmes', the demonstration that two different pairSCFG implementations can fold RNAs as well as the best current thermodynamic approach (Dynalign), despite the fact that our pairSCFGs clearly lack a treatment of some more complex statistical features known to be important in RNA structure, indicates that it will be promising to explore this direction further, with more biologically realistic grammars.

Parameterization of these grammars is also an area of future work. Thus far, we have used a single point estimate for our parameters, based on maximum a posteriori training using a mixed ribosomal RNA dataset. The most glaring problem with this is that since we are comparing RNA sequences of different levels of evolutionary divergence, we would prefer not to use single point estimates, but to instead use a rate-based model that allows our parameters to be conditional on evolutionary time. Preliminary data (not shown) shows that evolutionary models akin to those described by Knudsen and Hein [11,23] improves our performance relative to any point-estimated set of parameters.

## Authors' contributions

RDD developed the constrained Sankoff algorithm, wrote the code, carried out the experiments, and drafted the manuscript. Both authors collaborated closely in writing the final version.

## Appendix

The proposed grammar is both structurally unambiguous and alignment unambiguous. Reeder et. al. [50] suggested the following test for ambiguity.

We start from the observation that if a deterministic parser can be generated for a grammar, the grammar must be unambiguous by definition [51]. This observation alone is not useful, because the whole point of an RNA folding grammar requires it to be formally ambiguous in the sense that any given sequence has many possible structures (parse trees), not just one; the job of the folding algorithm is to find the optimal structure/parse tree. However, we can separate this necessary kind of ambiguity from the two types of undesired ambiguity (structural ambiguity, when the same base-paired structure corresponds to multiple parse trees; and alignment ambiguity, when the same set of aligned residue pairs corresponds to multiple parse trees). We redefine the grammar terminals such that the grammar emits an *annotation of the consensus structural alignment*, rather than an aligned sequence pair, such that each possible structural alignment has one and only one annotation string. Then, for this alternative representation of the grammar, we show that we can generate a deterministic parser.

For a single sequence, a nested (non-pseudoknotted) secondary structure is uniquely described by an annotation of unpaired ('.') and paired ('<', '>') bases. Extending this to structural alignment, we must also introduce gap symbols to indicate when an unpaired residue is only present in X ('-') or only present in Y ('_'). In our grammars, a consensus base pair must be present in both sequences, so gaps may only be placed in unpaired columns. Using these symbols, each structural alignment is uniquely described by a single annotation string. Figure 1 shows an example annotation string.

Because the rules of our pairSCFG directly map to the symbols (and meanings) of the annotation alphabet, we can transform our pairSCFG grammar into the following annotation grammar:

*S *→ .*S *| -*L*_*x *_| _*L*_*y *_| *T *| *ε*

*T *→ *T*. | *R*_*x*_- | *R*_*y*__ | <*P *> | *T *<*P *>

*L*_*x *_→ .*S *| -*L*_*x *_| *T *| *ε*

*L*_*y *_→ .*S *| _*L*_*y *_| *T *| *ε*

*R*_*x *_→ *T*. | *R*_*x*_- | <*P *> | *T *<*P *>

*R*_*y *_→ *T*. | *R*_*y*__ | <*P *> | *T *<*P *>

*P *→ <*P *> | *N*

*N *→ .*S *| -*L*_*x *_| _*L*_*y *_| *T*. | *R*_*x*_- | *R*_*y*__ | *T *<*P *>

Now, if a parser can be generated for this annotation grammar, then our pairSCFG must be unambiguous. Note that a failure to identify a parser would not prove that the grammar is ambiguous, but rather that no conclusion can be made with respect to the grammar's ambiguity status. The popular yacc and bison parse generators are only capable of handling a subset of context-free grammars and fail to generate a parser for the annotation grammar. A more general parse generator is the MSTA parse generator of the COCOM compiler construction package. The MSTA parse generator produces a parser for the annotation grammar, which demonstrates that our pairSCFG is structurally unambiguous and alignment unambiguous.
